# Accuracy of Different Lens Power Calculation Formulas in Patients With Mature Cataracts

**DOI:** 10.7759/cureus.47053

**Published:** 2023-10-15

**Authors:** Elif Ceren Yesilkaya, Ruveyde Garip

**Affiliations:** 1 Ophthalmology, University of Health Sciences, Sisli Hamidiye Etfal Research and Training Hospital, Istanbul, TUR; 2 Ophthalmology, Trakya University Faculty of Medicine, Edirne, TUR

**Keywords:** refractive errors, anterior chamber depth, lens thickness, mature cataract, intraocular lens power calculation formulas

## Abstract

Introduction

To compare the prediction accuracy of lens power calculation formulas in patients undergoing mature cataract surgery.

Methods

A total of 90 operations involving the Alcon SA60AT IOL implant (Alcon, Geneva, Switzerland) were analyzed in terms of mean refractive prediction error (PE) and mean absolute prediction error (MAE) using backward calculation in a retrospective design.

Results

A negative PE was observed in SRK/T, Holladay 1, Holladay 2, Hoffer Q, Haigis, and Emmetropia Verifying Optical (EVO) formulas. In contrast, positive PEs were observed in Barrett Universal II (BAUII), Kane, and Radial Basis Function (RBF) formulas. Negative PE was observed with all formulas, except BAUII, in patients with a shallow anterior chamber depth (ACD). While the SRK/T, Holladay 1, BAU, Kane, and RBF formulas demonstrated positive PE, the Holladay 2, Hoffer Q, Haigis, and EVO formulas indicated negative PE. In patients with deep ACD, positive PE was observed in all formulas, barring Holladay 2 and EVO. No significant differences were identified between the formulas concerning MAE and percentages of 0.25 diopter (D), 0.50 D, 0.75 D, and 1.0 D across all study groups.

Conclusion

Although the new generation formulas provide very good results, achieving the best with a single formula is still impossible.

## Introduction

Cataract, which is one of the most common causes of treatable blindness all over the world, is opacification of the lens material [1,2]. Cataracts in which the nucleus and cortex become too opaque to obstruct the red fundus reflex are called mature cataracts [2,3]. Nowadays, with the increase in individuals with active lifestyles, patients' refractive expectations after surgery have also increased and transformed cataract surgery into a refractive procedure [4]. Considering that postoperative refractive errors are the most common cause of dissatisfaction among patients, it is essential to achieve the target refraction by implanting the most accurate intraocular lens (IOL) for the patient. Thus, it is mandatory to calculate the accurate IOL power via the most suitable formula [5]. Frequently used IOL power formulas are the vergence-based formulas, including Barrett Universal II (BAUII) [6], Holladay 1 and 2 [7], Haigis [8], Hoffer Q [9], and SRK-T [10]. These formulas aim to estimate the effective lens position using various ocular biometric measurements such as anterior chamber depth (ACD), axial length (AL), and keratometry (K) readings. On the other hand, the ocular parameters can be affected by the degree of lens opacity. Moreover, in their study, Ueda T et al. found that the density of cataracts could influence the postoperative refractive results. They reported that higher cataract density might lead to inaccuracies in AL measurements, ultimately leading to less favorable postoperative refractive outcomes [11]. Currently, there is only a limited number of articles in the literature that have presented refractive data of mature, dense cataracts. The aim of this study was to compare the prediction accuracy of several new generation formulas (BAUII, Emmetropia Verifying Optical (EVO), Radial Basis Function (RBF)) and traditional formulas (Haigis, Hoffer Q, Holladay 1 and 2, and SRK/T) in patients undergoing mature cataract surgery and to investigate the effect of ACD and lens thickness (LT) measurements by IOLMaster 700 on IOL calculation in different formulas.

## Materials and methods

This retrospective, cross-sectional case series was conducted with 90 eyes of 90 patients who underwent uneventful cataract surgery with monofocal IOL implantation after a diagnosis of mature cataract. This study was carried out in accordance with the tenets of the Declaration of Helsinki and approved by the local Human Research and Ethics Committee of Trakya University (approval no. 05/28).

Study population

Ninety eyes of 90 patients older than 40 years of age who underwent uncomplicated phacoemulsification and monofocal IOL implantation with the diagnosis of mature and hard-dense cataracts were included in the study. Mature cataract was defined as highly opacified lens material (cortical, nuclear, or both) obscuring the red reflex [3].

The study's inclusion criteria and methodology adhered to the recommendations outlined in a recent study regarding protocols for assessing the accuracy of IOL formulas [11]. The exclusion criteria included cases with incomplete biometric data, LT measurements less than 2.50 mm, corneal astigmatism exceeding 3.0 diopters (D), a history of prior surgical procedures, complex phacoemulsification procedures, additional interventions during cataract surgery (e.g., combined vitrectomy), instances of postoperative complications, AL ≤20 mm and ≥25 mm, ultrasound biometric measurements, postoperative best-corrected visual acuity (BCVA) worse than 20/40, and inadequate documentation. The refraction examination performed in the first month after surgery was recorded for all patients.

Surgical procedure

All operations were performed by a single surgeon using the Constellation® vision system (Alcon, Geneva, Switzerland) through a 2.8 mm clear corneal temporal incision. All patients were implanted with the Alcon SA60AT IOL (Alcon, Geneva, Switzerland).

Data collection

Demographic characteristics, preoperative biometric measurements, IOL power, preoperative and postoperative best corrected visual acuities (BCVAs), manifest refraction, and spherical equivalent (SE) were recorded for each patient.

The same experienced technician made all preoperative biometric measurements using IOL Master®700 version 1.80.10.61129 (Carl Zeiss Meditec, Jena, Germany). Biometric measurements include AL, ACD, flat and steep K values, LT, and horizontal corneal diameter, also known as white to white (WTW). The lens power was calculated with the SRK-T formula. All measurements were confirmed with at least three consistent measurements.

The patients were divided into three groups according to LT percentiles as follows: Group 1 encompassed those with an LT of 4.13 or less; Group 2 included individuals with an LT between 4.13 and 4.74; and Group 3 consisted of those with an LT equal to or greater than 4.74.

The patients were divided into three groups according to preoperative ACD measurement also as follows: those with a shallow anterior chamber were defined as having an ACD of 3.00 mm or less; a regular anterior chamber was defined as an ACD greater than 3.00 mm but less than 3.50 mm; and a deep anterior chamber was defined as having an ACD of 3.50 mm or more.

Formula calculations

SE predictions from nine IOL power calculation formulas (SRK-T, Haigis, Holladay 1 and 2, Hoffer Q, BUII, Kane, EVO, and Hill RBF) were obtained using a keratometric index of 1.3375. The IOL Master 700 had a licensed version of SRK-T, Hoffer Q, Haigis, Holladay 1, and 2 formulas. Optimized A constant for SRK-T, pACD for Hoffer Q, a0, a1, and a2 for Haigis, and surgeon factor for Holladay suggested by User Group for Laser Interference Biometry (ULIB) [11] were used.

The BAUII formula was accessed online at https://calc.apacrs.org/barrett_universal2105/, and the Kane formula was available at https://www.iolformula.com/. The EVO formula was accessed online at https://www.evoiolcalculator.com/ and utilized a constant for SRK-T by ULIB [12], as recommended by the online calculator. The Hill RBF formula was accessed online at https://rbfcalculator.com/online/index.html. 

Constant optimization was conducted by the authors to zero out the arithmetic mean error by adjusting the refractive prediction error for each eye, either upward or downward, by an amount equal to the arithmetic mean error, as recommended by Wang L et al. [13].

Formula evaluation

The mean refractive prediction error (PE), mean absolute prediction error (MAE), median absolute prediction error (MedAE), and percentage of eyes with prediction error within ± 0.25 D, ± 0.50 D, ± 0.75 D, and ± 1.00 D were analyzed for each formula with backward calculation. The observed postoperative refraction was converted to SE, and the PE was calculated as the difference between refractive outcome and predicted SE. A negative PE value indicated a tendency toward overcorrection that would produce a more myopic result than intended, and vice versa. MAE is defined as the average of the absolute values (without considering the prediction error's sign). MedAE is defined as the midpoint value of absolute prediction error distribution.

Statistical analysis

Biometric and demographic data of study patients were presented with frequencies (percentages), mean ± SD, median, and interquartile range. Data normality was assessed by the Shapiro-Wilk test. The Quartile Split method was used to divide numerical data into three categories for LT. The one-sample t-test or the Wilcoxon signed-rank test was used to evaluate whether the mean refractive PE of each formula was significantly different from zero. Depending on the distribution pattern, the one-way ANOVA and Kruskal-Wallis tests were applied to compare data from the LT and ACD groups. Statistical analysis was performed using SPSS version 21.0 (IBM Corp., Armonk, NY, USA). A p-value of <0.05 was considered significant.

## Results

Demographic and biometric data

The total study population's demographic features and biometry data are listed in Table 1. The mean postoperative SE was -0.46 ± 0.80 D (between -3.63 to +1.50 D). Postoperative SE was found to be within ±1.00 D in 67.6% of the cases. The percentages of eyes achieving absolute errors within the dioptric ranges of 0.25 D, 0.50 D, 0.75 D, and 1.0 D of predicted refraction are presented in Table 2, and there was no significant difference between all IOL formulas in terms of percentages of each D (p>0.05). 

**Table 1:** The baseline characteristics of the entire study population. n: Number; BCVA: Best corrected visual acuity; mm: Millimeter; μm: Micrometer; K: Keratometry; D: Diopters; IOL: Intraocular lens.

	Mean ± SD	Median	Range
Age (years)	69.27 ± 10.17	69	42-89
Male gender, n (%)	49 (54.4)		
Preoperative BCVA (Snellen values)	0.006 ± 0.007	0.001	0.001-0.016
Axial length (mm)	23.38 ± 0.59	23.39	22.23-24.84
K1 (D)	42.95 ± 1.52	43.12	37.97-47.43
K2 (D)	44.03 ± 1.54	43.88	40.98-51.00
Anterior chamber depth (mm)	3.06 ± 0.38	3.08	2.29-3.82
Lens thickness (mm)	4.51 ± 0.52	4.51	3.29-5.88
Central corneal thickness (μm)	548.64 ± 32.81	546	489-635
Implanted IOL power (D)	22.31 ± 1.63	22.50	18.5-27.5
Postoperative spherical equivalent (D)	-0.46 ± 0.80	-0.50	-3.63-1.50
Postoperative BCVA (Snellen values)	0.79 ± 0.27	0.9	0.5-1.0

**Table 2:** Refractive prediction errors, SD, mean prediction error, and median absolute prediction error of each formula. PE: Prediction error; MAE: Mean absolute prediction error; MedAE: Median absolute prediction error; BAUII: Barrett Universal II; EVO: Emmetropia verifying optical; RBF: Radial basis function.

Formula	PE	SD	MAE	MedAE	The percentage spherical equivalent of the eyes (%)				
					≤±0.25	≤±0.50	≤±0.75	≤±1.00	˃1.00
SRK/T	0.0002	0.791	0.569	0.360	41.2	58.8	72.1	79.4	20.6
Holladay1	-0.014	0.819	0.603	0.455	32.3	54.4	69.1	77.9	22.1
Holladay2	-0.111	0.804	0.601	0.425	33.8	52.9	67.6	80.9	19.1
Hoffer Q	-0.066	0.861	0.644	0.545	20.6	45.6	72.1	79.4	20.6
Haigis	-0.049	0.859	0.632	0.477	32.3	51.5	69.1	77.9	22.1
BAUII	0.136	0.799	0.594	0.424	33.8	51.5	72.1	79.4	20.6
EVO	-0.031	0.792	0.602	0.442	30.9	55.9	72.1	77.9	22.1
KANE	0.035	0.822	0.617	0.480	32.3	51.5	67.6	80.9	19.1
RBF	0.003	0.785	0.579	0.398	36.8	57.3	72.1	82.4	17.6

Formula accuracy according to LT

The mean LT of the shallow, regular, and deep ACD groups were 4.89 ± 0.39 mm, 4.36 ± 0.39 mm, and 3.98 ± 0.44 mm, respectively. The mean refractive PE of each formula according to LT is shown in Table 3. All of the formulas had mean PE that were significantly different from zero. A negative PE was observed in SRK/T, Holladay 1, Holladay 2, Hoffer ˃Q, Haigis, and EVO, while positive PEs were observed in BAUII, Kane, and RBF formulas. In subgroup evaluations, positive PE was observed in all formulas in patients with LT of 4.13-4.74 mm, and negative PE was observed in patients with LT of ≥4.74 mm. In group 1 (LT≤4.13), BAUII and Kane formulas showed positive PE compared to other formulas. However, no significant correlation was found between the formulas and the LT measurements (p>0.05).

**Table 3:** Prediction error, SD, and median absolute error for subgroups in all formulas. BAUII: Barrett Universal II; EVO: Emmetropia verifying optical; RBF: Radial basis function; PE: Prediction error; MAE: Mean absolute prediction error; ACD: Anterior chamber depth. Group 1: Lens thickness (LT) ≤4.13, Group 2: LT between 4.13 and 4.74, Group 3: LT ≥4.74. Shallow ACD: ≤3.00mm, Regular ACD: 3.00 mm <ACD<3.50 mm, Deep ACD: ≥3.5 mm.

	SRK/T	Holladay1	Holladay2	Hoffer Q	Haigis	BAUII	EVO	Kane	RBF
Group 1 (n=16)									
PE	0.413	0.520	0.461	0.599	0.583	0.540	0.445	0.501	0.498
SD	0.355	0.465	0.442	0.547	0.560	0.500	0.411	0.479	0.468
MAE	0.320	0.339	0.239	0.461	0.485	0.376	0.276	0.380	0.363
Group 2 (n=38)									
PE	0.530	0.547	0.557	0.547	0.559	0.525	0.528	0.543	0.508
SD	0.449	0.419	0.420	0.408	0.406	0.421	0.367	0.368	0.397
MAE	0.370	0.474	0.444	0.556	0.520	0.429	0.446	0.565	0.412
Group 3 (n=36)									
PE	0.704	0.670	0.675	0.685	0.654	0.695	0.715	0.683	0.641
SD	0.683	0.671	0.690	0.672	0.671	0.627	0.615	0.652	0.630
MAE	0.390	0.484	0.416	0.594	0.420	0.429	0.439	0.482	0.398
Shallow ACD (n=44)									
PE	0.717	0.708	0.699	0.731	0.705	0.688	0.732	0.740	0.693
SD	0.669	0.659	0.640	0.651	0.646	0.597	0.581	0.600	0.603
MAE	0.532	0.571	0.685	0.588	0.532	0.525	0.531	0.554	0.565
Regular ACD (n=35)									
PE	0.515	0.563	0.570	0.582	0.592	0.558	0.538	0.542	0.692
SD	0.438	0.465	0.452	0.500	0.527	0.488	0.437	0.481	0.603
MAE	0.373	0.421	0.455	0.525	0.410	0.363	0.364	0.373	0.338
Deep ACD (n=11)									
PE	0.367	0.411	0.361	0.482	0.446	0.377	0.365	0.382	0.389
SD	0.298	0.327	0.325	0.375	0.361	0.298	0.276	0.249	0.300
MAE	0.308	0.285	0.240	0.403	0.318	0.330	0.266	0.349	0.355

Formula accuracy according to the ACD

The mean AL for the shallow ACD, regular ACD, and deep ACD groups were 23.27 ± 0.67 mm, 23.40 ± 0.63 mm, and 23.78 ± 0.53 mm, respectively. A significant difference was observed in AL values between the shallow and deep ACD groups (p=0.005), as well as between the regular and deep ACD groups (p=0.042). In the shallow ACD group, all formulas exhibited a negative PE, with the exception of BAUII. In patients with regular ACD, while SRK/T, Holladay 1, BAU, Kane, and RBF demonstrated a positive PE, Holladay 2, Hoffer Q, Haigis, and EVO exhibited a negative PE. In the deep ACD group, a positive PE was observed in all formulas except for Holladay 2 and EVO. A significant positive correlation was found between the PE error of the Hoffer Q formula and ACD (r=0.253, p=0.02), whereas no significant correlation was identified between ACD and other formulas (p>0.05). No significant differences were observed between the formulas in terms of MAE across all study groups (p=0.101). In the subgroup analysis based on LT and ACD, no differences were found between the MAE values of the formulas (p>0.05). In patients with shallow ACD, the Kane, EVO, Holladay II, and SRK/T formulas showed significantly higher MAE values compared to those in the deep ACD group (respectively; p=0.017, p=0.009, p=0.035, and p=0.027). However, no significant differences were observed between the three groups in terms of the BAUII, Haigis, Hoffer Q, Holladay 1, and Hill-RBF formulas (respectively; p=0.055, p=0.191, p=0.198, p=0.092, and p=0.059).

## Discussion

Predicting the postoperative refractive outcome of cataract surgery is essential for the ophthalmologist, and choosing the right IOL power can be more challenging in cases such as mature cataracts. The main findings of our study demonstrated that a negative PE was observed in SRK/T, Holladay 1, Holladay 2, Hoffer Q, Haigis, and EVO, while positive PEs were observed in BAUII, Kane, and RBF formulas. According to LT, while positive PE was observed in all formulas in patients with LT of 4.13-4.74 mm, negative PE was observed in patients with LT of ≥4.74 mm. In patients with LT≤4.13 mm, BAUII, and Kane formulas showed positive PE compared to other formulas. In all formulas, it was observed that there was a tendency to shift to hyperopia in thin lenses and to myopia in thick lenses. In contrast to our findings, Hipólito-Fernandes D et al. reported that there was a tendency towards hyperopia in thick lenses and myopia in thin lenses [14]. This was interpreted as the thicker lens predicting higher dioptric power due to the decreased ACD in mature cataract patients and, therefore, the deepened anterior chamber and lens position after surgery, causing myopia. In the shallow ACD group, negative PE was observed in all formulas except BAUII. While SRK/T, Holladay 1, BAUII, Kane, and RBF showed positive PE, Holladay 2, Hoffer Q, Haigis, and EVO showed negative PE in patients with regular ACD. Positive PE was observed in the deep ACD group in all formulas except Holladay 2 and EVO. When considering the arithmetic PE based on LT percentiles from 10th to 90th, regardless of ACD, and comparing our findings with Melles RB et al., a consistent trend emerged across all formulas. We observed a tendency for a myopic shift with thinner lenses and a hyperopic shift with thicker lenses, with the Haigis formula showing this pattern most prominently [15]. Furthermore, we noted that Hill-RBF V.2.0 was also notably influenced by LT, exhibiting a PE distribution remarkably similar to that of the Haigis formula. In line with Melles RB et al.'s study, the BAUII formula exhibited higher PE (indicating a hyperopic shift) with greater LT values when compared to the Holladay 1 and Hoffer Q formulas [15]. However, it is essential to approach comparisons with Melles RB et al.'s study cautiously, as our investigation exclusively encompassed eyes with AL falling between 22.0 and 26.00 mm, with a focus on a comprehensive analysis within this "regular" subset of eyes where IOL formulas are expected to perform optimally. Notably, the PEARL-DGS and Kane formulas consistently yielded more stable results across LT variations. Although studies comparing IOL formulations are frequent in routine cases, limited studies evaluate conditions such as mature cataracts. Leng L et al., in their study, evaluated the effect of cataract progression on IOL power measurements. They found no difference between K and AL evaluations between the two measurements in biometry measurements performed at least three months before and one day before the surgery. However, they found a significant difference between ACD and IOL power [16]. In addition, in this case, two measurements were statistically significant in terms of MAE. Therefore, measurements made without increased cataract density may reflect more accurate results in patients followed for cataract progression. Ueda T et al. found that postoperative refractive outcome is affected by cataract density and that an increase in cataract density may cause errors in AL measurement, suggesting that this may lead to worse postoperative refractive outcomes [17]. According to Freeman G et al., measurement failure is frequently caused by mature and posterior subcapsular cataracts at a rate of 15.9% [18]. Similarly, in our study, we found that 20.6% of the patients had a refractive error greater than 1 D.

Tabatabaei SA et al., in their study, evaluated frequently used IOL formulations in patients with phacomorphic glaucoma three months after cataract surgery [19]. The study revealed significant differences in PE among the five formulas assessed. The Holladay I formula exhibited the least error (-0.02 ± 1.11). In contrast, the Haigis formula demonstrated the highest hyperopic shift (0.37 ± 1.22), the highest MAE (0.99 ± 0.78), and the lowest rates of achieving the desired PE outcomes. Conversely, the SRK II formula achieved the highest percentages of desired PEs. It is worth noting that the overall differences in MAE among the five formulas did not reach statistical significance. Specifically, the Haigis formula yielded positive mean PE values, indicating a greater hyperopic shift than predicted, while the other formulas resulted in myopic mean PEs, suggesting that the IOL was positioned more anteriorly than anticipated. This observation implies that older-generation IOL power calculation formulas may yield more favorable refractive outcomes, possibly due to the inherent unpredictability of postoperative ACD. Our study observed that the best results were in the SRK/T and RBF formulas when looked at according to the PE values. Again, regarding MAE and MedAE values, it was seen that SRK/T, RBF, and BAUII formulas were determined more accurately. However, no statistically significant difference was found with other formulas.

According to Zhu X et al., the amount of lens opacity was associated with poor preoperative biometric fixation stability, resulting in AL errors that ultimately lead to postoperative refractive errors [20]. Jeong J et al. tried to measure how various biometric factors affect the accuracy of IOL formulas [21]. When assessing mean refractive error, effective lens position (ELP) and changes in ACD at one month after surgery were statistically significantly associated with the Holladay 1, Hoffer Q, and SRK/T formulas but not with the Haigis formula. K values and preoperative ACD were not associated with errors in any of the formulas mentioned previously. The authors concluded that the Haigis formula may be the most accurate option when ACD shifts are expected, such as in patients with mature cataracts, zonular weakness, or preoperative angle-closure glaucoma.

Hipólito-Fernandes D et al., in their study, examined the effect of ACD and LT values on the accuracy of nine different IOL power calculation formulas in patients with normal AL [14]. If ACD is ≤3.0 mm and ≥3.5 mm, SRK/T, Hoffer Q, and Holladay I found that the mean PE was significantly different from 0. However, BAUII, Kane, PEARL-DGS, and EVO V.2.0 were more accurate than Hoffer Q in ACD ≤3.0 mm. They found that BAUII, Kane, PEARL-DGS, and EVO V.2.0 revealed the lowest mean and MedAE variance regarding the ACD and LT subgroups. In their study, Haigis and Hill-RBF V.2.0 were significantly impacted by LT, independent of ACD, with a myopic shift with thin lenses and a hyperopic shift with thick lenses. In our study, when examining the MAE and MedAE values, SRK/T, Holladay 2, EVO, and RBF produced the best results in the group with LT <4.13. In contrast, the most accurate results in the group with LT >4.74 were observed with RBF, Haigis, Holladay, and Kane formulas. However, this difference was not statistically significant compared to other formulas. Additionally, BAUII and EVO formulas provided the most accurate results in terms of MAE and MedAE in the group with ACD<3.00 mm. Holladay formulas and EVO provided the most accurate results in the group with ACD>3.50 mm. No statistical difference was found with other formulas. Considering these results, the group that was least affected by LT and ACD was the RBF and EVO formula, although this difference was not statistically significant with other formulas. In the patients with a SE value below 0.50 D, the highest accuracy rates were also observed in the SRK/T, RBF, and EVO formulas, although no statistically significant difference was noted between the formulas.

Our study limitations include a relatively small sample size and limited range of AL, so it was impossible to generalize the results to all AL ranges. To our knowledge, this is the first study to evaluate the effect of LT and ACD values on the accuracy of old and new-generation formulas in mature cataract patients. Therefore, it does not seem possible to make a critical comparison of our results with the published literature on this subject. Further studies are required with a larger sample size and inclusion of all AL ranges.

## Conclusions

The present study was conducted to investigate the effect of mature cataracts on the accuracy of IOL power measurement. Although the new generation formulas provide excellent results, achieving the best with a single formula is still impossible. This issue arises because questions, such as whether the ACD was directly affected by LT, which results from a thickened lens, or whether a shallow ACD is genuinely an anatomically shallow ACD, still cannot be precisely determined by the formulas.
